# The Association Between Loss of Consciousness and the Default Mode Network at the Chronic Stage of Diffuse Axonal Injury: A Diffusion Tensor Imaging Study

**DOI:** 10.3390/jcm15187205

**Published:** 2026-09-17

**Authors:** Sung Ho Jang, Min Jye Cho, Dong Hyun Byun

**Affiliations:** 1 Department of Physical Medicine and Rehabilitation, College of Medicine, Yeungnam University, Namku, Daegu 42415, Republic of Korea; strokerehab@hanmail.net (S.H.J.); ctfmon00617@daum.net (M.J.C.); 2 Department of Special Creative Convergence, College of Rehabilitation Sciences, Daegu University, Jillyang-Eup, Gyeongsan-si 38453, Republic of Korea

**Keywords:** default mode network, loss of consciousness, diffuse axonal injury, diffusion tensor tractography, diffusion tensor imaging

## Abstract

**Highlights:**

**What are the main findings?**

- After controlling for clinical confounders, the duration of loss of consciousness (LOC) exhibits a marginal trend toward a negative correlation with the tract volume (TV) of the mPFC-PCC/precuneus neural connectivity in patients with diffuse axonal injury (DAI).
- The LOC duration shows a marginal trend toward a positive correlation with the mean diffusivity (MD) of the MTL-RSC neural connectivity.

**What are the implications of the main findings?**

- Chronic-stage microstructural changes in the default mode network (DMN) are likely influenced by several factors, including the severity of the initial injury and the time since injury, rather than by the LOC duration alone.
- Although the LOC duration provides useful clinical information, it should not be considered a direct predictor of chronic DMN deterioration in patients with DAI.

**Abstract:**

**Objectives**: We investigate the relationship between the loss of consciousness (LOC) and the state of default mode network (DMN) connectivity in diffuse axonal injury (DAI) patients, using diffusion tensor tractography (DTT). **Methods**: Twenty-one consecutive patients with DAI were recruited in this study. The DMN connectivity [medial prefrontal cortex (mPFC)—posterior cingulate cortex (PCC)/precuneus and retrosplenial cortex (RSC)—medial temporal lobe (MTL)] was reconstructed using DTT. Fractional anisotropy (FA) value, mean diffusivity (MD) value, and tract volume (TV) of the DMN connectivity at the chronic stage were measured in this study. **Results**: After adjusting for covariates, none of the DTT parameters reached definitive statistical significance at the *p* < 0.05 level. However, the LOC duration revealed a marginal trend toward a negative correlation with the TV of the mPFC-PCC/precuneus neural connectivity (partial *r* = −0.466, *p* = 0.060) and a marginal trend toward a positive correlation with the MD value of the MTL-RSC neural connectivity (partial *r* = 0.467, *p* = 0.059). The FA and MD of the mPFC-PCC/precuneus, and the FA and TV of the MTL-RSC, showed no significant associations (*p* > 0.05). **Conclusions**: This exploratory study identified a marginal trend between the LOC duration and the state of DMN connectivity (mPFC-PCC/precuneus and MTL-RSC) at the chronic stage in DAI patients. Chronic-stage microstructural changes in the DMN are likely influenced by several factors, including the severity of the initial injury and the time since injury. Therefore, although the LOC duration provides useful clinical information, it should not be considered a direct predictor of chronic DMN deterioration in patients with DAI.

## 1. Introduction

Traumatic brain injury (TBI) is one of the most common neurologic disorders causing disability, and impaired consciousness is one of the main sequelae [1]. Approximately 50% of all patients with severe head trauma suffer from diffuse axonal injury (DAI)—a common cause of unconsciousness and persistent vegetative state [2,3]. DAI occurs due to shearing forces from acceleration and deceleration, or rotational forces on the brain during accidents. It is characterized by widespread axonal injury and immediate loss of consciousness (LOC) lasting more than 6 h [4,5,6,7]. LOC, the state of altered alertness and awareness at onset, is an important indicator for classifying injury severity and predicting the prognosis of consciousness outcome in TBI patients [8,9,10]. Therefore, elucidating the neural status underlying the LOC is important for establishing rehabilitative strategies to restore consciousness in patients with impaired consciousness following DAI. However, very little is known about the relationship between the LOC and the state of the white matter structures in DAI.

In widespread axonal injury following DAI, the majority of diffuse axonal lesions are found in regions of the default mode network (DMN), such as the posterior cingulate cortex (PCC), retrosplenial cortex (RSC), precuneus, medial prefrontal cortex (mPFC), inferior parietal and posterior temporal areas, hippocampal formation, and medial temporal lobe (MTL), which are damaged by shearing forces [11,12,13,14]. The DMN plays a partial role in intrinsic awareness and is activated when individuals are not focused on external conditions, including during daydreaming and mind-wandering [15,16]. Among the components of the DMN, the connectivity between the mPFC and PCC/precuneus is known to be associated with internal consciousness and awareness [17,18,19,20]. On the other hand, the connectivity between the RSC and MTL is responsible for critical conjunctions in arousal and awareness, and conscious perception, respectively [21,22,23]. In addition, a strong concordance between resting-state functional and structural connectivity within the RSC-MTL network has been reported [21,22,23]. Therefore, confirming the state of DMN connectivity and its correlation with the LOC would be important for estimating impaired consciousness following DAI. Importantly, the microstructural alterations of the white matter in DAI are not limited to primary mechanical shearing but evolve into secondary time-dependent neurodegenerative processes, including Wallerian degeneration, demyelination, and axonal loss, particularly at the chronic stage [2,3,6].

Conventional brain computed tomography (CT) or magnetic resonance imaging (MRI) provides only a limited understanding of the DMN in a live human brain. Recently, diffusion tensor tractography (DTT), derived from diffusion tensor imaging (DTI), has enabled three-dimensional reconstruction of the DMN in the human brain [17,23]. Among the neuroimaging studies on the relationship of the DMN with the disorders of consciousness (vegetative state, minimally conscious state, and emerging from a minimally conscious state) using functional MRI(fMRI) and DTI [17,24,25,26,27,28,29,30,31,32,33,34,35,36], one study used DTT to demonstrate the association between the states of DMN connectivity (PCC/precuneus–temporoparietal junctions, PCC/precuneus–thalamus, temporoparietal junctions–thalamus) and consciousness using the Coma Recovery Scale-Revised (CRS-R) [17]. However, no study has examined the relationship between the state of the DMN and the LOC to date. Furthermore, chronic-stage microstructural alterations in the brain are highly multifactorial, heavily influenced not only by the initial injury severity markers, such as the initial Glasgow Coma Scale (GCS) score and post-traumatic amnesia (PTA) duration, but also by natural aging and the temporal interval since the trauma (time to DTI) [8,9]. Therefore, investigating simple unadjusted correlations may lead to biased structural interpretations. We hypothesized that the LOC duration is negatively associated with the state of DMN connectivity (mPFC-PCC/precuneus and MTL-RSC) in DAI patients within a complex multifactorial context. Consequently, this study aimed to investigate the independent relationship between the LOC duration and the state of DMN connectivity at the chronic stage in DAI patients using DTT, rigorously controlling for primary clinical confounding factors.

## 2. Methods

### 2.1. Subjects

Twenty-one consecutive patients with TBI (15 men, 6 women; mean age 36.10 ± 16.68 years; range, 20~66 years) were recruited according to the following inclusion criteria: (1) first-ever TBI; (2) age of 20–69 years; (3) LOC duration following trauma lasting six hours or more; (4) no specific brain lesions except for the DAI lesion (petechial white matter microhemorrhage or focal areas of gliotic scarring) on conventional MRI; (5) DTI scanning was performed at a chronic stage (more than one month after onset); (6) no previous history of head trauma and psychiatric and neurological disease [5,11,37]. Patients with persistent disorders of consciousness, including coma, vegetative, and minimally conscious states, were excluded. The demographic and clinical data of the patients are shown in Table 1. Furthermore, to maximize data transparency and reproducibility, the detailed individual profiles of all 21 patients are provided in Supplementary Table S1. This study was performed retrospectively, and the study protocol was approved by Yeungnam university hospital (Protocol code YUMC 2021-03-014-005).

### 2.2. Diffusion Tensor Imaging and Tractography

DTI data were acquired at an average of 10.47 ± 13.66 months after the onset of head trauma. DTI was performed using a sensitivity-encoding head coil on a 1.5 T Philips Gyroscan Intera (Hoffman-LaRoche Ltd., Best, The Netherlands) scanner with single-shot echo-planar imaging and navigator echo. Sixty contiguous slices (acquisition matrix = 96 × 96; reconstruction matrix = 192 × 192; field of view = 240 mm × 240 mm; Repetition Time (TR) = 10,726 ms; Echo Time (TE) = 76 ms; b = 1000 s/mm^2^; Number of Excitation (NEX) = 1; and thickness = 2.5 mm) were acquired for each of the 32 noncollinear diffusion-sensitizing gradients. Image distortion due to eddy current and head motion effects was corrected using affine multi-scale two-dimensional registration at the Oxford Centre for Functional Magnetic Resonance Imaging of Brain (FMRIB) Software Library (FSL v6.0.5: https://fsl.fmrib.ox.ac.uk/fsl/docs/. accessed on 2 November 2021) [38]. Fiber tracking was performed using the tractography routines implemented in the FMRIB Diffusion Toolbox (https://fsl.fmrib.ox.ac.uk/fsl/fslwiki/FDT. accessed on 2 November 2021) to apply a probabilistic tractography method based on a multi-fiber model (5000 streamline samples, 0.5 mm step lengths, curvature threshold  =  0.2) [39,40]. To track neural connectivity between the mPFC and the PCC/precuneus, the seed region of interest (ROI) was manually located on the PCC and precuneus in a coronal view at the same coordinate [18]. Target ROIs were placed on the anterior cingulate cortex and mPFC (Brodmann’s areas 14, 24, 25, and 32) in the coronal view. The superior boundary of the ROI in the mPFC was the cingulate sulcus; the medial boundary was the midline between the right and left hemispheres. The lateral boundary was a line 11.25 mm lateral from the midline [41]. For reconstruction of neural connectivity between the MTL and the RSC, the seed and target ROIs were manually placed in coronal view on the MTL and the RSC (Brodmann’s areas 29 and 30), respectively [23,42]. All fibers stretched out into the contralateral hemisphere were excluded. Of the 5000 samples produced in the seed voxel, results for positive contacts were visualized at a minimum threshold of 2 for the DMN connectivity. Fractional anisotropy (FA) value, mean diffusivity (MD) value, and tract volume (TV) of the DMN connectivity were measured in this study.

### 2.3. Statistical Analysis

Statistical analysis was performed using SPSS 21.0 for Windows (SPSS, Chicago, IL, USA). The Shapiro–Wilk test was performed to determine the normality of the LOC duration and DTT (FA, MD, and TV) parameters of the DMN connectivity. The LOC duration, the TV of the mPFC-PCC/precuneus neural connectivity, and the FA value of the MTL-RSC neural connectivity did not satisfy the assumption of normality (*p* < 0.05). Therefore, to rigorously investigate the independent relationships between the LOC duration and the DTT parameters while accounting for potential confounding factors, a non-parametric partial correlation analysis was employed. Prior to the partial correlation analysis, all continuous variables were converted into rank-ordered data (rank transformation). The analysis was then performed controlling for age, initial Glasgow Coma Scale (GCS) score, post-traumatic amnesia (PTA) duration, and time from trauma to DTI as covariates. A *p*-value of <0.05 was considered statistically significant, and *p*-values between 0.05 and 0.10 were defined as indicating a marginal trend toward significance. The partial correlation coefficient (*r*) represents the directionality (positive or negative) and strength of the independent relationship between two variables (0.10~0.29: weak correlation; 0.30~0.49: moderate correlation; more than 0.50: strong correlation) [43].

## 3. Results

A total of 21 consecutive patients with DAI (males: 15, females: 6; mean age: 36.10 ± 16.68 years, range: 20–66 years) were included in this study. The clinical characteristics of the patients are summarized in Table 1 (and detailed in Supplementary Table S1). The mean initial GCS score at the time of injury was 7.14 ± 3.38 (range: 3–15). The mean duration of post-traumatic amnesia (PTA) was 27.48 ± 24.47 days. The mean LOC duration across all patients was 15.92 ±17.40 days (range: 0.31–56 days). DTI scanning was performed at a mean of 10.47 ± 13.66 months (range: 1.43–57.83 months) after the onset of TBI.

Regarding the state of the DMN connectivity at the chronic stage, the DTT parameters were successfully reconstructed and measured for all 21 patients (Table 2). For the mPFC-PCC/precuneus neural connectivity, the mean FA value was 0.31 ± 0.03, the mean MD value was 0.89 ± 0.06, and the mean TV was 1347 ± 920. For the MTL-RSC neural connectivity, the mean FA, MD, and TV values were 0.27 ± 0.03, 1.12 ± 0.1, and 730 ± 208, respectively.

**Figure 1:**
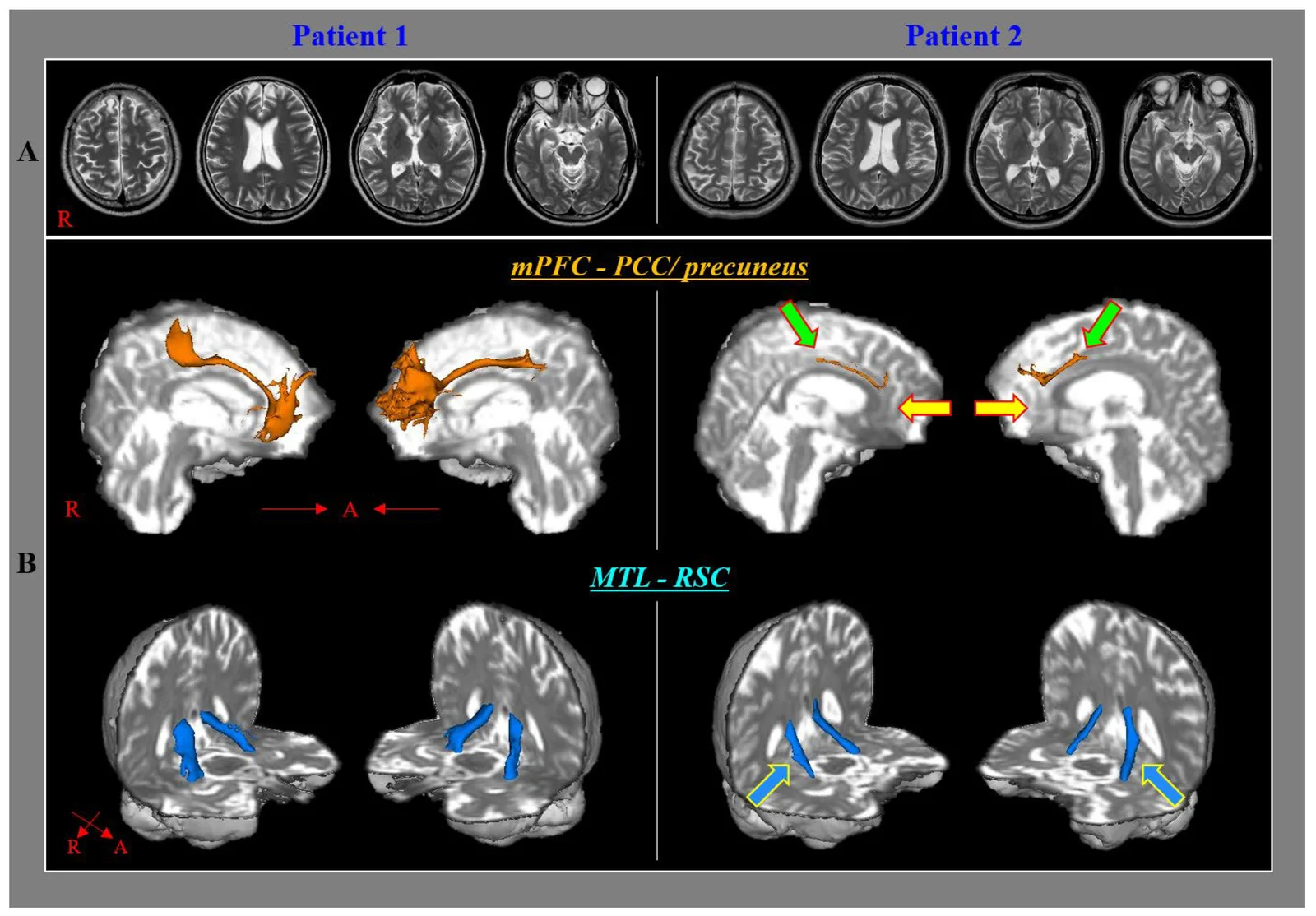
Results of diffusion tensor tractography for the default mode network (DMN) connectivity [medial prefrontal cortex (mPFC)—posterior cingulate cortex (PCC)/precuneus, medial temporal lobe (MTL)—retrosplenial cortex (RSC)]. (**A**) T2-weighted brain magnetic resonance images at the time of diffusion tensor imaging scanning in representative patients with a diffuse axonal injury at the chronic stage [patient 1: 45-year-old male with four days of loss of consciousness (LOC) duration, patient 2: 23-year-old male with 30 days of LOC duration]. (**B**) The mPFC—PCC/precuneus neural connectivity in patient 2 shows discontinuation near the mPFC and PCC/precuneus (green and yellow arrows). In addition, narrowing fibers of the MTL—RSC neural connectivity in patient 2 are reconstructed (sky blue arrow) compared with those in patient 1.

The partial correlations between the LOC duration and DTT parameters of the patients are listed in Table 3. After strictly controlling for age, initial GCS score, PTA duration, and time to DTI using a non-parametric partial correlation analysis, none of the DTT parameters reached statistical significance at the *p* < 0.05 level. However, the LOC duration exhibited a strong marginal trend toward a negative correlation with the TV of the mPFC-PCC/precuneus neural connectivity (partial *r* = −0.466, *p* = 0.060). In addition, a marginal trend toward a positive correlation was observed between the LOC duration and the MD value of the MTL-RSC neural connectivity (partial *r* = 0.467, *p* = 0.059). Other parameters, including the FA and MD of the mPFC-PCC/precuneus and the FA and TV of the MTL-RSC, showed no significant associations (*p* > 0.05). These microstructural alterations underlying the marginal trends can be visually observed in the representative DTT images (Figure 1).

## 4. Discussion

In this study, DTT was conducted to investigate the independent association between the LOC duration and the state of DMN connectivity at the chronic stage in DAI patients, controlling for critical clinical confounders. The results can be summarized as follows: after adjusting for age, initial GCS score, PTA duration, and time to DTI, the LOC duration showed a marginal trend toward a positive correlation with the MD values of the MTL-RSC neural connectivity, and a marginal trend toward a negative correlation with the TV of the mPFC-PCC/precuneus neural connectivity. Among the most commonly used DTT parameters, the FA value reflects the integrity and directional organization of white-matter microstructures by quantifying the degree of directional diffusion of water molecules [44,45,46]. The MD value, which indicates the magnitude of water diffusion within brain tissue and thus reflects the loss of myelin and axons, is commonly used to detect pathological changes and neuronal injury in the white matter [47,48,49]. The TV is determined by the number of voxels occupied by all streamlines within the neural structure and reflects the total number of neural fibers [50]. The absence of a significant correlation of the duration of LOC with the MD value of the mPFC—PCC/precuneus neural connectivity and TV of the MTL—RSC neural connectivity suggests that the duration of LOC was not associated with the axonal and myelin loss of the mPFC—PCC/precuneus neural connectivity, and with the number of remaining neural fibers of the MTL—RSC neural connectivity.

In this study, upon strictly controlling for initial injury severity (GCS, PTA) and the time variable (time to DTI), the relationships between the LOC duration and the MD value of the MTL-RSC neural connectivity (positive) and the TV of the mPFC-PCC/precuneus neural connectivity (negative) demonstrated marginal trends rather than definitive statistical significance. These marginal trends imply that prolonged LOC duration may partially reflect the trajectory of axonal injury in the MTL-RSC and fiber loss in the mPFC-PCC/precuneus; however, the LOC duration alone cannot be interpreted as a direct, isolated cause of chronic DMN deterioration. Considering the functions of the above connectivity areas, the LOC duration might be associated with arousal, awareness, and conscious perception via the MTL-RSC neural connectivity, and impact internal consciousness and awareness via the mPFC-PCC/precuneus neural connectivity, within a complex multifactorial context. The absence of significant correlations between the LOC duration and the FA values of DMN connectivity could suggest that the FA value was unaltered. However, there were potentially significant changes in the underlying tissues because the DAI could damage all the underlying fiber bundles within a particular region equally [i.e., equal reduction in three eigenvalues (λ1, λ2, and λ3)] [51].

Several studies have used DTI to investigate the relationship between the duration of LOC and various white matter structures, including the brainstem, uncinate fasciculus, inferior frontal–occipital fasciculus, and the ascending reticular activating system, in TBI patients [52,53,54]. In 2015, Delano-Wood et al. demonstrated associations between the LOC duration and FA values of the corticospinal tract in the lower brainstem in chronic-stage mild-to-moderate TBI patients [54]. In 2016, Wilde et al. reported the correlations of the LOC duration with the FA value (negative) of the uncinate fasciculus and the MD values (positive) of the uncinate fasciculus and inferior frontal occipital fasciculus at the acute stage in mild TBI patients [52]. In 2019, Jang et al. demonstrated that LOC duration was related to the FA values (negative) of the lower ventral and upper ascending reticular activating system, and the TV (negative) of the lower dorsal ascending reticular activating system at more than half a month after onset in TBI patients [53]. Based on the above studies, to the best of our knowledge, this is the first study to report the relationships between the LOC duration and the state of DMN connectivity (mPFC-PCC/precuneus and MTL-RSC) in DAI patients using DTT.

Nevertheless, this study had several limitations. First, false-positive or false-negative results in fiber tract reconstruction could arise from crossing fibers or partial-volume effects during DTT analysis [55]. Second, in the clinical setting, sedation or endotracheal intubation during the acute stage of TBI could limit the accurate estimation of LOC duration. Third, there is potential for referral bias, as the clinical features may be more severe than those in the general population of DAI patients at the chronic stage, as patients who visited the university hospital’s rehabilitation department were recruited for this study. Finally, the relatively small sample size (*n* = 21) coupled with the inclusion of multiple clinical covariates in the partial correlation analysis may have constrained the statistical power, potentially resulting in the observation of marginal trends rather than definitive statistical significance. Hence, further prospective studies considering these limitations are necessary. Future longitudinal studies combining functional MRI and post-TBI DTT are warranted to reveal the selective vulnerability of neural tracts. Moreover, incorporating advanced molecular imaging, such as F18 or Cu62/64-labeled ligands tracking mitochondrial dynamics, could provide a crucial bridge between preclinical TBI models [56,57] and clinical neuroimaging data, thereby focusing targeted rehabilitative treatments.

In conclusion, this exploratory study identified a marginal trend between the LOC duration and the state of DMN connectivity (mPFC-PCC/precuneus and MTL-RSC) at the chronic stage in DAI patients. The current findings underscore that chronic DMN deterioration is a highly multifactorial process, heavily influenced by the initial injury severity and recovery time, rather than solely determined by the LOC duration. Therefore, while estimating the LOC duration provides valuable clinical context, establishing it as a definitive prognostic biomarker for DMN connectivity requires further validation through large-scale, prospective studies.

## Figures and Tables

**Table 1:** The demographic and clinical data of the patients.

	Patients
Age (years)	36.10 ± 16.68
Number (*n*)	21
Male:Female	15:6
LOC duration (days)	15.92 ±17.40
PTA duration (days)	27.48 ± 24.47
GCS score	7.14 ± 3.38
DTI duration (months)	10.47 ± 13.66

LOC: loss of consciousness, PTA: post-traumatic amnesia, GCS: Glasgow coma scale, DTI: diffusion tensor imaging; values are mean ± standard deviations.

**Table 2:** The diffusion tensor tractography parameters of the patients.

mPFC—PCC/Precuneus			MTL—RSC		
FA	MD	TV	FA	MD	TV
0.31 ± 0.03	0.89 ± 0.06	1347 ± 920	0.27 ± 0.03	1.12 ± 0.1	730 ± 208

mPFC: medial prefrontal cortex, PCC: posterior cingulate cortex, MTL: medial temporal lobe, RSC: retrosplenial cortex, FA: fractional anisotropy, MD: mean diffusivity, TV: tract volume; values are mean ± standard deviations.

**Table 3:** Correlations between the loss of consciousness and diffusion tensor tractography parameters of the patients.

		mPFC—PCC/Precuneus			MTL—RSC		
		FA	MD	TV	FA	MD	TV
LOC duration	*r*-value	0.150	−0.03	−0.466	−0.108	0.467	0.243
	*p*-value	0.566	0.908	0.060	0.681	0.059	0.347

mPFC: medial prefrontal cortex, PCC: posterior cingulate cortex, MTL: medial temporal lobe, RSC: retrosplenial cortex, FA: fractional anisotropy, MD: mean diffusivity, TV: tract volume, LOC: loss of consciousness. Partial correlation coefficients (*r*) and (*p*)-values were calculated using a non-parametric partial correlation analysis (following rank transformation) controlling for age, initial Glasgow Coma Scale score, post-traumatic amnesia duration, and time to diffusion tensor imaging.

## Data Availability

Data are available on request due to privacy/ethical restrictions.

## Supplementary Materials

The following supporting information can be downloaded at https://www.mdpi.com/article/10.3390/jcm15187205/s1, Table S1: Demographic and clinical data of individual patients with diffuse axonal injury. This table provides the detailed individual profiles of all 21 patients included in the study.
