# Supplementary material for: The Association Between Loss of Consciousness and the Default Mode Network at the Chronic Stage of Diffuse Axonal Injury: A Diffusion Tensor Imaging Study
Source: J Clin Med. 2026 Sep 17;15(18):7205. doi: 10.3390/jcm15187205 (PMC13607626; doi:10.3390/jcm15187205)
Supplement: Supplementary file 1 [file jcm-15-07205-s001.zip › jcm-4493957-supplementary.pdf]

Table S1: Demographic and clinical data of individual patients with diffuse axonal injury

							mPFC - PCC / precuneus			MTL-RSC		
	AGE (years)	SEX	PTA duration (days)	GCS score	DTI duration (months)	LOC duration (days)	FA	MD	TV	FA	MD	TV
Patient 1	22	F	7	15	6.8	0.31	0.36	0.81	1045	0.37	0.99	238.5
Patient 2	20	M	15	8	1.47	2	0.3	0.91	1671.5	0.24	1.07	999.5
Patient 3	24	F	3	8	1.73	3	0.32	0.87	311	0.25	1.15	912.5
Patient 4	22	M	15	8	1.5	4	0.31	0.86	1385.5	0.31	0.97	581
Patient 5	45	M	10	13	15.93	4	0.28	1.01	3561	0.24	1.1	750
Patient 6	24	M	4	5	2.4	4	0.33	0.84	2076	0.29	0.96	900
Patient 7	25	M	14	7	2.3	5	0.32	0.9	2764	0.3	1.02	835
Patient 8	23	M	7	7	1.43	5	0.26	1	3124.5	0.25	1.05	862.5
Patient 9	31	F	21	8	13.57	7	0.33	0.83	1433	0.25	1.21	890.5
Patient 10	65	F	20	6	6.97	7	0.28	0.96	1599.5	0.33	1.19	434
Patient 11	64	M	14	8	25.43	7	0.3	0.91	1252.5	0.28	1.01	735.5
Patient 12	28	M	28	8	6.87	10	0.3	0.93	835	0.25	1.23	549.5
Patient 13	33	M	10	3	9.13	10	0.41	0.78	389.5	0.31	1.1	973
Patient 14	63	M	10	9	2.4	10	0.34	0.85	1525	0.26	1.16	1006.5
Patient 15	26	M	70	3	8.83	20	0.35	0.85	1665	0.23	1.15	797.5
Patient 16	55	F	56	3	4.37	21	0.33	0.87	411	0.32	1.01	465.5
Patient 17	66	F	21	5	1.63	28	0.24	1.01	974	0.23	1.3	806.5
Patient 18	23	M	56	4	13.3	30	0.29	0.96	301	0.25	1.16	612.5
Patient 19	35	M	56	3	32.63	45	0.28	0.94	900.5	0.25	1.18	728
Patient 20	21	M	84	6	3.37	56	0.33	0.85	329	0.27	1.3	479
Patient 21	43	M	56	13	57.83	56	0.33	0.92	751.5	0.23	1.24	774

mPFC: medial prefrontal cortex, PCC: posterior cingulate cortex, MTL: medial temporal lobe, RSC: retrosplenial cortex, PTA: post-traumatic amnesia, GCS: Glasgow coma scale, DTI: diffusion tensor imaging, LOC: loss of consciousness, FA: fractional anisotropy, MD: mean diffusivity, TV: tract volume,
